# Asset pricing with long-run disaster risk

**DOI:** 10.1371/journal.pone.0287687

**Published:** 2023-06-27

**Authors:** Rujie Fan, Chao Xiao

**Affiliations:** School of Finance, Southwestern University of Finance and Economics, Chengdu, Sichuan, China; Università Cattolica del Sacro Cuore Sede di Piacenza e Cremona Facoltà di Economia: Universita Cattolica del Sacro Cuore Facolta di Economia e Giurisprudenza, ITALY

## Abstract

Traditional disaster models with time-varying disaster risk are not perfect in explaining asset returns. We redefine rare economic disasters and develop a novel disaster model with long-run disaster risk to match the asset return moments observed in the U.S. data. The difference from traditional disaster models is that our model contains the long-run disaster risk by treating the long-run ingredient of consumption growth as a function of time-varying disaster probability. Our model matches the U.S. data better than the traditional disaster model with time-varying disaster risk. This study uncovers an additional channel through which disaster risk affects asset returns and bridges the gap between long-run risk models and rare disaster models.

## Introduction

Traditional rare disaster models play an essential role in explaining asset returns [1–10]. At present, there are three main methods for modeling disaster risk: (i) the static disaster probability (a constant) [2, 4, 6, 11]; (ii) the exogenous time-varying disaster probability (e.g., a square-root process) [8, 12–15]; (iii) the endogenous time-varying disaster probability (e.g., a Bayesian learning process) [16–24]. These studies have shown that the time-varying disaster risk, rather than the static disaster risk, is the key to successfully interpreting asset returns by traditional disaster models. However, traditional disaster models with time-varying disaster risk are far from perfect in explaining asset returns.

In this paper, we construct a new discrete-time disaster model to explain asset returns in the United States. We assume that the long-run ingredient in consumption growth rates is a function of the time-varying disaster probability. Consumption growth faces the standard diffusion risk, the time-varying disaster risk, and the long-run disaster risk; the latter two are two manifestations of disaster risk. A disaster is modeled as a jump with a negative size. Meanwhile, we assume that the jump follows a Bernoulli distribution, the jump size follows a gamma distribution, and time-varying disaster probability obeys a square-root process [25]. Using the technique of linearity-generating processes [26], our model is tractable, and all asset prices are solved in closed form.

The solution for our model reveals that the time-varying disaster risk alone is insufficient to generate the equity premium, the volatility of equity returns, and the Sharpe ratio observed from the U.S. data. The model fits well with the data by combining time-varying disaster risk with long-run disaster risk. Adding the long-run disaster risk to the traditional time-varying disaster framework not only comprehensively improves the model’s ability to explain asset returns but also closely links rare disaster models with long-run risk models. Moreover, compared to the traditional time-varying disaster model, our model more strongly implicates equity premiums’ predictability and consumption growth’s unpredictability. All traditional disaster pricing models imply that consumption growth is predictable in long horizons. However, consumption growth is unpredictable in the data, especially in long horizons. The long-run disaster risk can mitigate such conflicts because it has a much smaller impact on consumption growth than on the price-dividend ratio.

Few studies combine the long-run risk with the disaster risk to explain asset returns. Bansal et al. [27] propose a temperature-augmented long-run risk model in which the long-run ingredient in consumption growth rates is subject to standard stochastic shocks and natural disasters caused by climate change. In contrast, we assume that the long-run ingredient is a linear function of time-varying disaster probability following a square-root process [25]. Barro and Jin [28] build a model with rare disasters and long-run risk and assume that the probability of disasters is constant and that the long-run ingredient in consumption growth rates is consistent with that in Bansal and Yaron [29]. Their assumption lacks strong links between traditional disaster risk and long-run risk. In our model, the dynamics of both the jumps (disasters) and the long-run ingredient depend on time-varying disaster probability. There is also some other literature related to the assumptions in the theoretical model of this paper. According to Barro and Jin [30], the distribution of transformed sizes of disasters, as defined by Barro and Ursúa [2], fits well with a power-law density. However, according to our definition of disasters, the disaster sizes tend to follow a gamma distribution. Similar to this paper, Bansal et al. [27] and Marfè and Pénasse [12] also assume that the disaster size follows a gamma distribution. To explain aggregate stock market volatility, unlike the existing literature on models with Bayesian learning or a Markov process [3, 16, 24, 30, 31], Wachter [8] assumes that the time-varying disaster probability obeys a square-root process [25]. Wachter [8] does not introduce long-run risk in the theoretical model; while its model can generate higher stock return volatility than dividend volatility, it also leads to excessive volatility in consumption and dividends.

## Rare economic disasters

As in Marfè and Pénasse [12], we define a rare economic disaster as a severe decline in consumption during a given year. A country experiences a rare economic disaster in the year *t* + 1 if Δ*c*_*t*+1_ falls by two times the standard deviation (*SD*) from its growth path:

$$N_{t+1}=\left\{\begin{array}{l} 1,\;\;\;\;\;|\;\;if\;\Delta c_{t+1}<mean(\Delta c)-2\times SD(\Delta c)\\ 0,\;\;\;\;|\;\;otherwise \end{array}\right.,$$ (1)

where Δ*c*_*t*+1_ = log *C*_*t*+1_ ‒ log *C*_*t*_, and *N*_*t*+1_ = 1 means an economic disaster with a disaster size of *mean* (Δ*c*) ‒ Δ*c*_*t*+1_ occurred in period *t* + 1.

We use long-term real per capita consumption expenditure data from 42 countries to assess historical economic disasters. Among them, the data on real consumption expenditure per capita before 2006 is provided by Barro and Ursúa [2]. We refer to Barro and Ursúa [2] to expand the data to 2021 and calculate the consumption growth rate from the consumption data. Descriptive statistics on the growth rate of real consumption expenditure per capita by country and the distribution of rare economic disasters on the timeline are included in detail in S1 Data. The sample size of the growth rate of real consumption expenditure per capita is equal to 5348. Table 1 presents descriptive statistics of rare economic disasters. The number of disasters is 183, the probability of a disaster is 0.0342, the average size of disasters is 0.1827, and the standard deviation of disaster sizes is 0.0907.

**Table 1:** Descriptive statistics of rare economic disasters.

Number of Disasters	183
Average Disaster Probability	0.0342
Average Disaster Size	0.1827
Std Dev Disaster Size	0.0907

**Fig 1:**
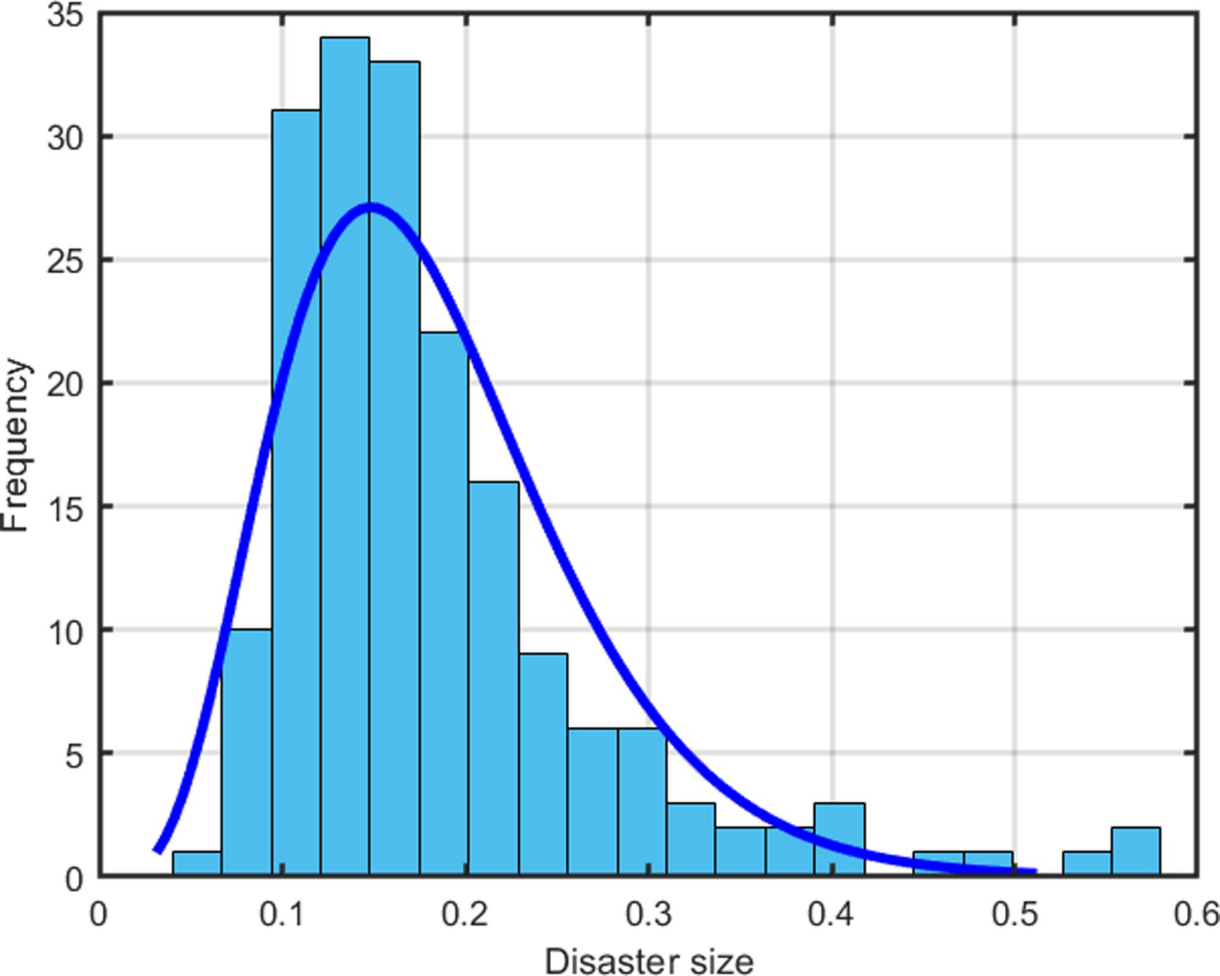
Distribution of disaster sizes.

**Fig 2:**
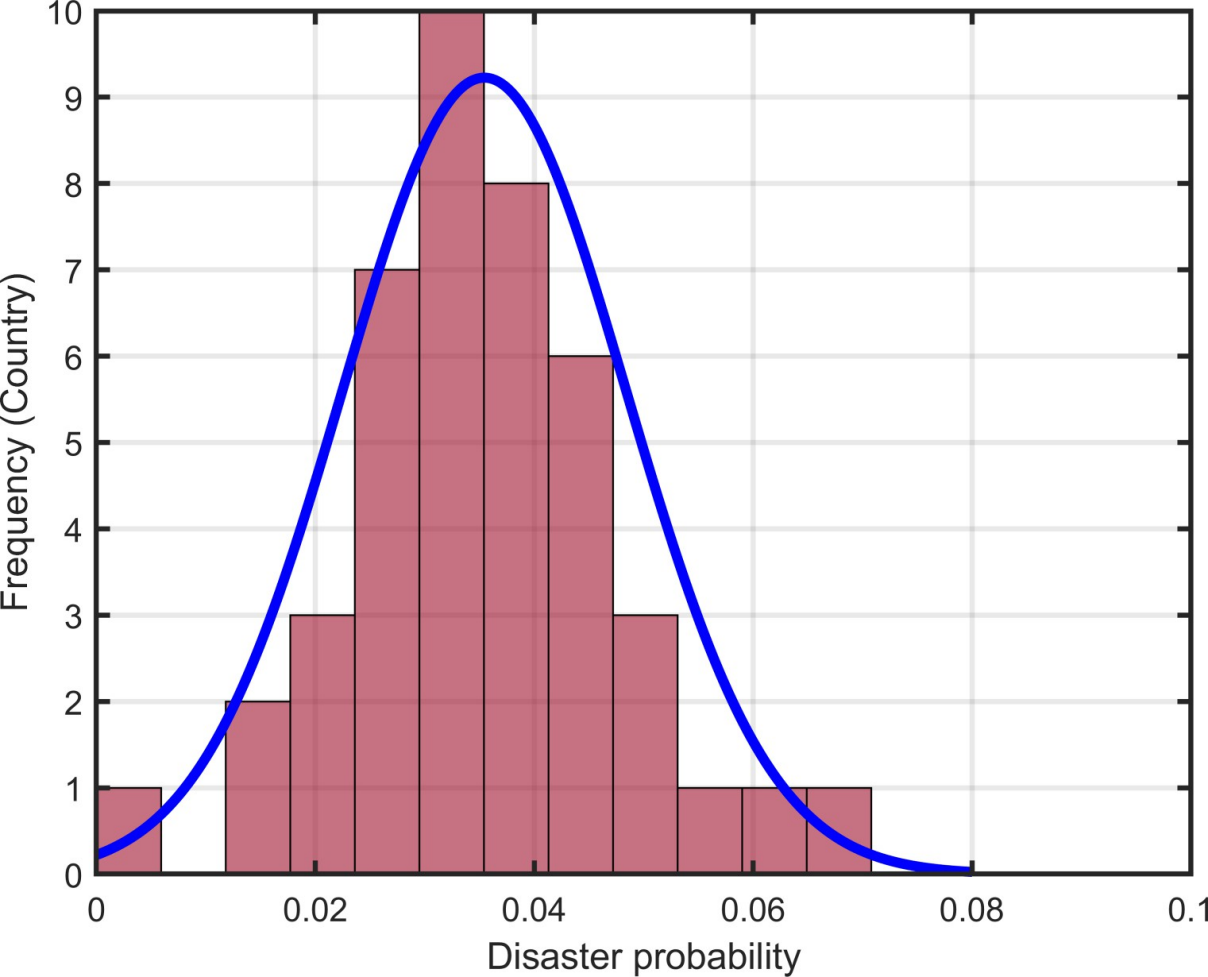
Distribution of disaster probability.

Next, we continue to study the distribution characteristics of disaster sizes. Barro and Jin [30] argue that the distribution of transformed sizes of disasters fits well with a power-law density. However, as shown in Fig 1, the Gamma density rather than the power-law density better fits the size distribution of our disasters. Fig 2 shows the distribution of disaster probabilities close to the normal distribution.

## The model

Agents have a recursive but not time-separable preference [32, 33]:

$$U_{t}={\left[(1-\delta ){\left(C_{t}\right)}^{1-1/\psi }+\delta E_{t}{\left[{\left(U_{t+1}\right)}^{1-\gamma }\right]}^{\frac{1-1/\psi }{1-\gamma }}\right]}^{\frac{1}{1-1/\psi }},$$ (2)

where variables *U*_*t*_ and *C*_*t*_ represent the utility and consumption of agents, δ is the subjective discount factor, and *ψ* and γ measure the elasticity of intertemporal substitution and the relative risk aversion, respectively. Eq (2) suggests that agents are risk-averse about potential future consumption risk. In this paper, we refer to Wachter [8] and Vissing-Jørgensen [18] to set *ψ* in Eq (2) to one for tractability. It is worth noting that the reasonable value of *ψ* is controversial [8, 9, 18, 29, 34–38].

## Proposition 1

When *ψ* is equal to one, the agents’ preference is as follows:

$$\log U_{t}=(1-\delta )\log C_{t}+\frac{\delta }{1-\gamma }\log E_{t}\left[e^{(1-\gamma )\log U_{t+1}}\right].$$ (3)

The proof of proposition 1 is shown in Appendix A in S1 Appendix. Agents receive an endowment consumption stream *C*_*t*_. The log consumption growth rate is given by

$$\Delta c_{t+1}=\mu +\tau p_{t}+\sigma \varepsilon_{t+1}-\vartheta_{t+1}N_{t+1},$$ (4)

where Δ*c*_*t*+1_ = log *C*_*t*+1_ ‒ log *C*_*t*_,*μ* is the constant term, *σ* represents the volatility of consumption growth without disaster risk, and *ε*_*t*+1_ ∼ *N*(0,1). *N*_*t*+1_ follows a Bernoulli distribution:

$$N_{t+1}=\left\{\begin{array}{l} 1,\;\;\;\;\;\;\;\;\;\;\;\;p_{t}\\ 0,\;\;\;\;1-\;p_{t}{\;}^{,} \end{array}\right.$$ (5)

In Eq (5), *p*_*t*_ represents the disaster probability. *p*_*t*_ obeys a square-root process [25]:

$$p_{t+1}=\theta +\phi p_{t}+\sigma_{p}\sqrt{p_{t}}\varepsilon_{p,t+1},$$ (6)

where 1 ≥ *p*_*t*_ ≥ 0,θ is the constant term, *ϕ* represents the rate of mean reversion of disaster probability, *σ_p_* is the volatility parameter of disaster probability, and *ε*_*t*+1_ ∼ *N*(0,1). The unconditional mean of disaster probability is θ/(1‒ϕ), and its unconditional variance can be written as $\theta \sigma_{p}^{2}/\left[\left(1-\phi \right)\left(1-\phi^{2}\right)\right]$. Therefore, *τp*_*t*_ in Eq (4) means that the disaster probability has a long-run negative impact on consumption growth (*τ* < 0). In this paper, the AR(1) process of the long-run component of consumption growth is as follows:

$$\tau \left(p_{t+1}-\frac{\theta }{1-\phi }\right)=\tau \phi \left(p_{t}-\frac{\theta }{1-\phi }\right)+\tau \sigma_{p}\sqrt{p_{t}}\varepsilon_{p,t+1}.$$ (7)

Eq (4) can be rewritten as:

$$\Delta c_{t+1}=\mu +\frac{\tau \theta }{1-\phi }+\tau \left(p_{t}-\frac{\theta }{1-\phi }\right)+\sigma \varepsilon_{t+1}-\vartheta_{t+1}N_{t+1}.$$ (8)

In Eq (4), *ϑ*_*t*+1_ indicates the disaster size. We assume that it follows a gamma distribution Γ(λ,ι) with a moment-generating function given by

$$f(i)=E_{t}\left[e^{i\vartheta_{t+1}}\right]={\left(1-\frac{i}{l}\right)}^{-\lambda },$$ (9)

where the mean and variance are equal to *λ/ι* and *λ/ι*^2^, respectively.

## Asset return

### Stochastic discount factor

Eq (3) implies that the log stochastic discount factor is

$$m_{t+1}=\log\delta -\Delta c_{t+1}+(1-\gamma )\zeta_{t+1}-\log E_{t}\left(e^{(1-\gamma )\zeta_{t+1}}\right),$$ (10)

where *ζ*_*t*+1_ = *log U*_*t*+1_ –*log C*_*t*_. An affine expression for the log stochastic discount factor can be obtained by the following assumptions and propositions. We first give assumption 1.

#### Assumption 1

*ζ*_*t*+1_ = *ϖ*_*t*+1_ – ϑ_*t*+1_*N*_*t*+1_, where *ϖ*_*t*+1_ follows a normal distribution and is independent of *ϑ*_*t*+1_ *N*_*t*+1_, *E*_*t*_(*ϖ*_*t*+1) =_ *v*_*t*_, and $var_{t}\left(\varpi_{t+1}\right)=\chi_{t}^{2}$.

#### Proposition 2

According to Assumption 1, the following equation can be derived:

$$E_{t}\left[e^{(1-\gamma )\zeta_{t+1}}\right]=e^{(1-\gamma )v_{t}+\frac{{\left(1-\gamma \right)}^{2}}{2}\chi_{t}^{2}+\left[{\left(1-\frac{\gamma -1}{l}\right)}^{-\lambda }-1\right]p_{t}}.$$ (11)

The proof of proposition 2 is shown in Appendix B in S1 Appendix. Moreover, we give propositions 3 and 4.

#### Proposition 3

If we define *y*_*t*_ = log *U*_*t*_−log *C*_*t*_, then Eq (3) can be transformed into

$$y_{t}=\delta \left[v_{t}+\frac{1-\gamma }{2}\chi_{t}^{2}+\frac{{\left(1-\frac{\gamma -1}{l}\right)}^{-\lambda }-1}{1-\gamma }p_{t}\right].$$ (12)

#### Proposition 4

*y*_*t*_ = *L*_0_ + *Lp*_*t*_, where $L_{0}=\frac{2\delta \left(\mu +L\theta \right)+\delta \left(1-\gamma \right)\sigma^{2}}{2\left(1-\delta \right)}$, $L=\frac{1-\delta \phi -\sqrt{{\left(1-\delta \phi \right)}^{2}-2\delta^{2}\sigma_{p}^{2}\left[\left(1-\gamma \right)\tau +{\left(1-\frac{\gamma -1}{\iota }\right)}^{-\lambda }-1\right]}}{\delta \left(1-\gamma \right)\sigma_{p}^{2}}$. Meanwhile, we can write that *v*_*t*_ = *L*_0_ + *μ* + *Lθ* + (*Lϕ+ τ)p*_*t*_ and $\chi_{t}^{2}=\sigma^{2}+{\left(L\sigma_{p}\right)}^{2}p_{t}$.

Proofs for propositions 3 and 4 are shown in Appendices C and D, respectively. Therefore, Eq (10) can be rewritten as follows:

$$\begin{array}{l} m_{t+1}=\underset{standard\;model}{\underset{︸}{\log\delta -\mu -\frac{{\left(1-\gamma \right)}^{2}\sigma^{2}}{2}-\gamma \sigma \varepsilon_{t+1}}}+\\ \underset{our\;model\;with\;disaster\;risk}{\underset{︸}{\left\{1-{\left(1-\frac{\gamma -1}{\iota }\right)}^{-\lambda }-\frac{{\left[\left(1-\gamma \right)L\sigma_{p}\right]}^{2}}{2}-\tau \right\}p_{t}+\left(1-\gamma \right)L\sigma_{p}\sqrt{p_{t}}\varepsilon_{p,\;t+1}+\gamma \vartheta_{t+1}N_{t+1}}}. \end{array}$$ (13)

### The risk-free rate

The log risk-free rate $\left(r_{t}^{f}\right)$ satisfies

$$r_{t}^{f}=\underset{standard\;model}{\underset{︸}{\mu -\log\delta +\left(1-2\gamma \right)\sigma^{2}/2}}\;+\underset{our\;model\;with\;disaster\;risk}{\underset{︸}{\left[{\left(1-\frac{\gamma -1}{\iota }\right)}^{-\lambda }-{\left(1-\frac{\gamma }{\iota }\right)}^{-\lambda }+\tau \right]p_{t}}}.$$ (14)

**Fig 3:**
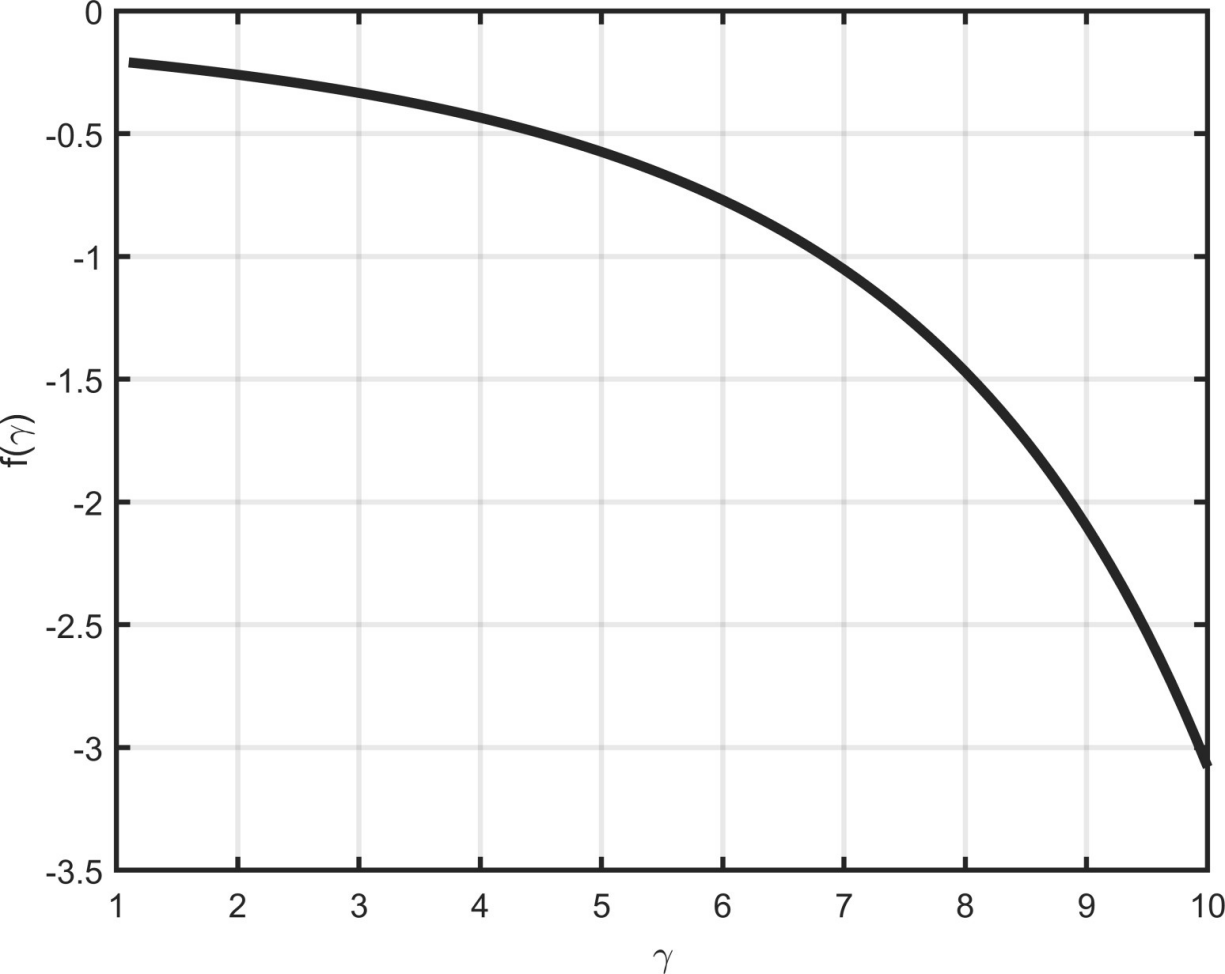
Relative risk aversion and disaster risk.

In the standard model without disaster risk, the log risk-free rate is expressed by the term above the first bracket in Eq (14); *μ* refers to the average growth rate of consumption without disaster risk; −log *δ* represents the rate of time preference; (1–2*γ*)*σ*^2^ / 2 stands for the effect of precautionary saving due to the standard risk (*σε*_*t*+1_) on the log risk-free rate. The term above the second bracket in Eq (14) is derived from disaster risk. In theory, because people are risk-averse, an increase in the probability of disasters will strengthen people’s willingness to save, thereby reducing the risk-free rate. Thus, if we define that $f\left(\gamma \right)={\left(1-\frac{\gamma -1}{\iota }\right)}^{-\lambda }-{\left(1-\frac{\gamma }{\iota }\right)}^{-\lambda },\;f\left(\gamma \right)$ should be negative. As shown in Fig 3, *f* (*γ*) decreases as risk aversion increases.

**Fig 4:**
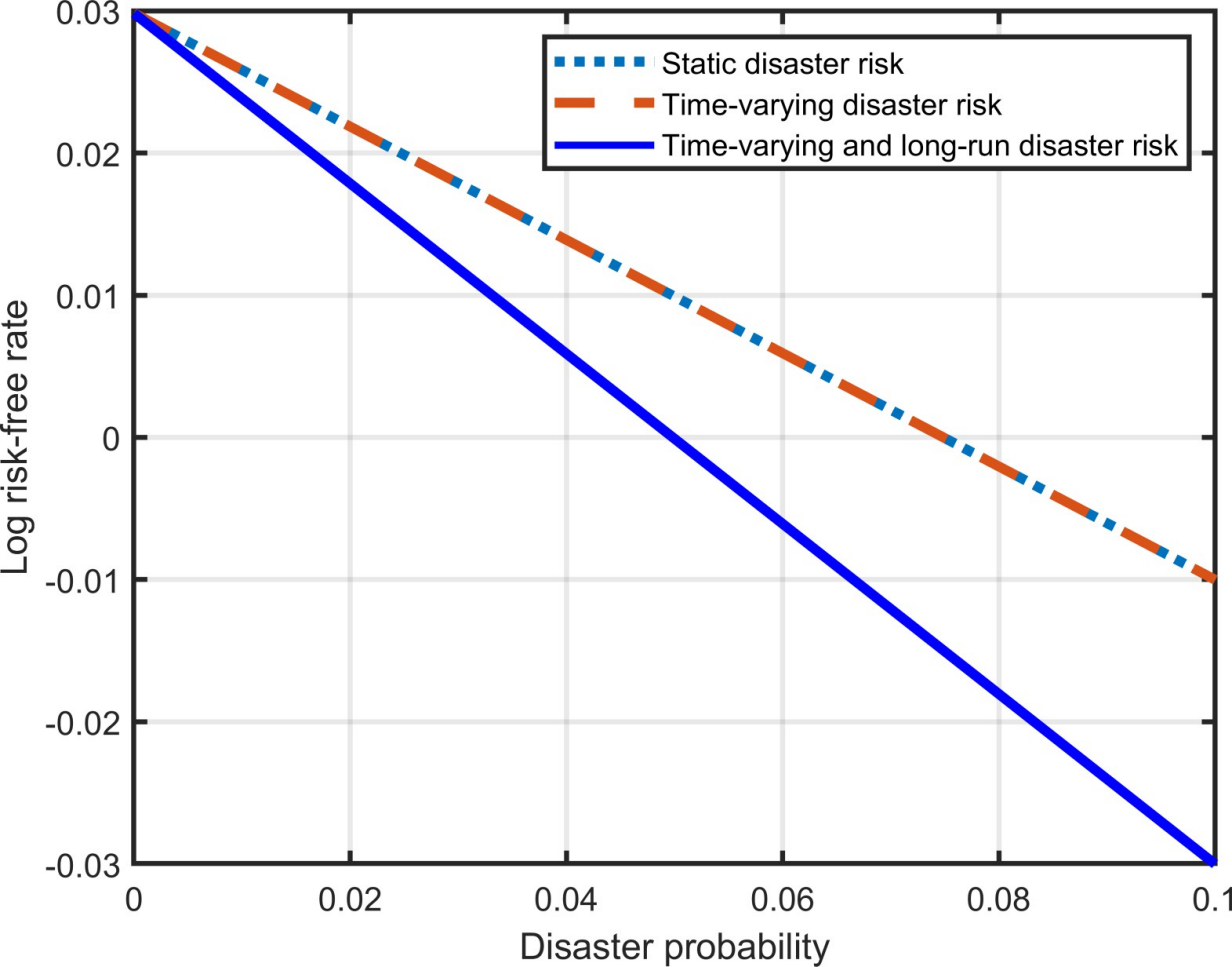
Effect of the long-run disaster risk on the risk-free rate.

**Fig 5:**
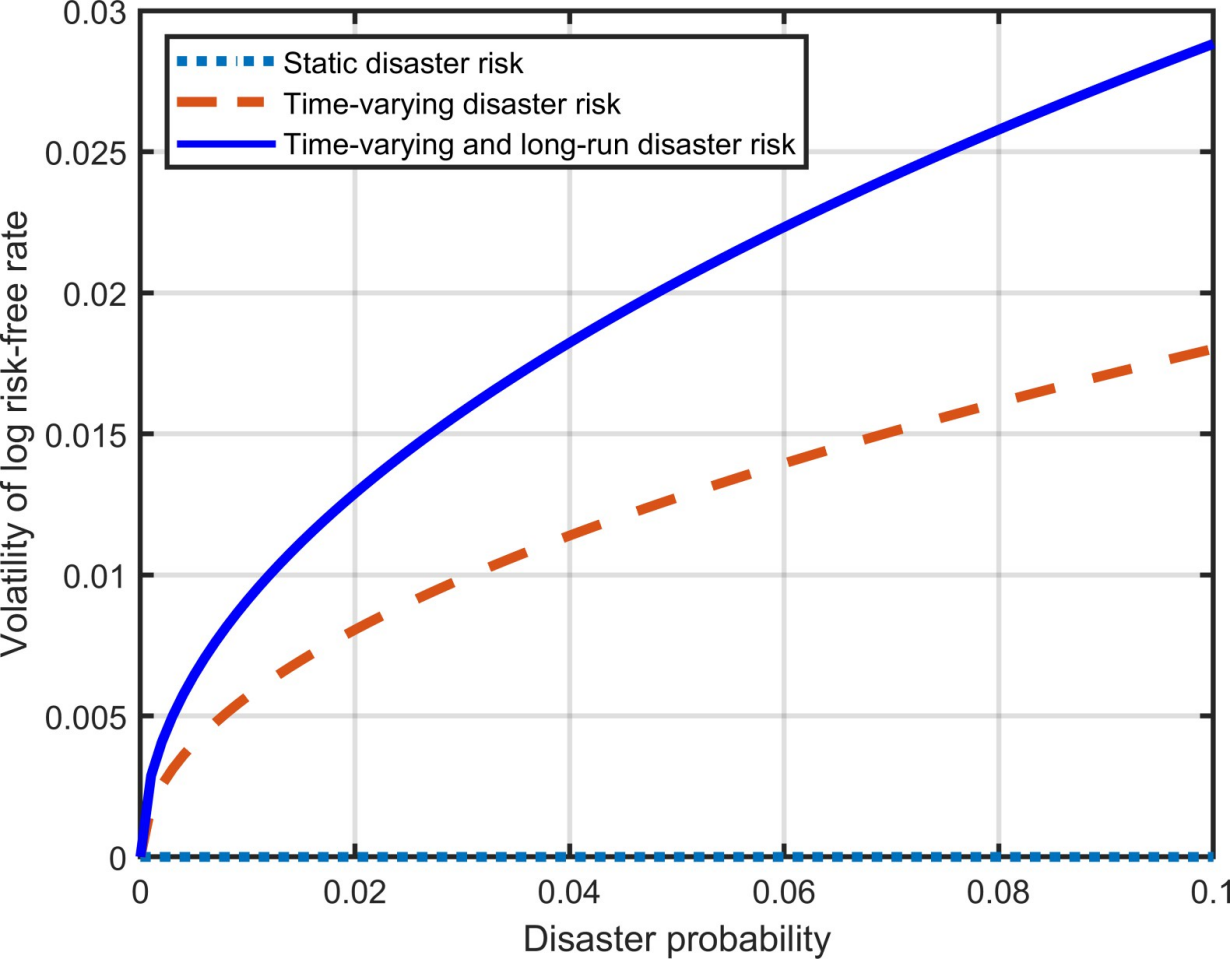
Effect of the long-run disaster risk on the volatility of the risk-free rate.

When is equal to zero, Eq (14) reflects the relationship between the risk-free rate and the disaster probability in traditional time-varying disaster risk models. As shown in Eq (8), we introduce the long-run disaster risk into consumption growth by setting *τ* not equal to zero. Therefore, both time-varying and long-run disaster risk are included in our model. Fig 4 shows that the risk-free rate is a decreasing function of disaster probability. The dashed line represents the traditional time-varying disaster risk model, while the solid line represents our model. When the disaster probability equals zero, the risk-free rates derived from the two models are the same. The solid line lies below the dashed line, as long-run disaster risk further boosts savings. The difference between the solid and dashed lines reflects the effect of long-run disaster risk on the risk-free rate. This effect increases significantly as the probability of disasters increases. Fig 5 indicates that the long-run disaster risk can increase the volatility of the risk-free rate.

### The equity return

Let Δ*d*_*t+*1_ denote the dividend growth rate. We model the dividend growth rate as a function of the consumption growth rate [8, 35, 39–41]:

$$\Delta d_{t+1}=\Phi \Delta c_{t+1}$$ (15)

where Φ is the leverage parameter. The equity return, *r*_*e*,*t*+1_, can be expressed in terms of the log price-dividend ratio (*pd*_*t*+1_ and *pd*_*t*_) as

$$r_{e,t+1}=\log\left(e^{pd_{t+1}}+1\right)-pd_{t}+\Delta d_{t+1}.$$ (16)

First, linearizing Eq (16) around the steady state of *pd*_*t*_ defined as *pd*_*ss*_, we obtain:

$$r_{e,t+1}\approx \log\left(e^{pd_{ss}}+1\right)-lpd_{ss}+lpd_{t+1}-pd_{t}+\Delta d_{t+1},$$ (17)

where $l=\frac{e^{pd_{ss}}}{e^{pd_{ss}}+1}$. According to proposition 5 below, we can obtain the affine expression of *r*_*e*,*t*+1_.

#### Proposition 5


$$pd_{t}=A_{0}+Ap_{t},$$ (18)

where $A_{0}\frac{\log\left[\delta \left(e^{pd_{ss}}+1\right)\right]-lpd_{ss}+\left(\Phi -1\right)\mu +l\theta A-\frac{{\left(1-\gamma \right)}^{2}\sigma^{2}}{2}+\frac{{\left(\Phi -\gamma \right)}^{2}\sigma^{2}}{2}}{1-l}$, $A=\frac{-\left[l\left(1-\gamma \right)L\sigma_{p}^{2}+l\phi -1\right]-\sqrt{{\left[l\left(1-\gamma \right)L\sigma_{p}^{2}+l\phi -1\right]}^{2}-2l^{2}\sigma_{p}^{2}\left[\left(\Phi -1\right)\tau +{\left(1-\frac{\gamma -\Phi }{\iota }\right)}^{-\lambda }-{\left(1-\frac{\gamma -1}{\iota }\right)}^{-\lambda }\right]}}{l^{2}\sigma_{p}^{2}}$.

**Fig 6:**
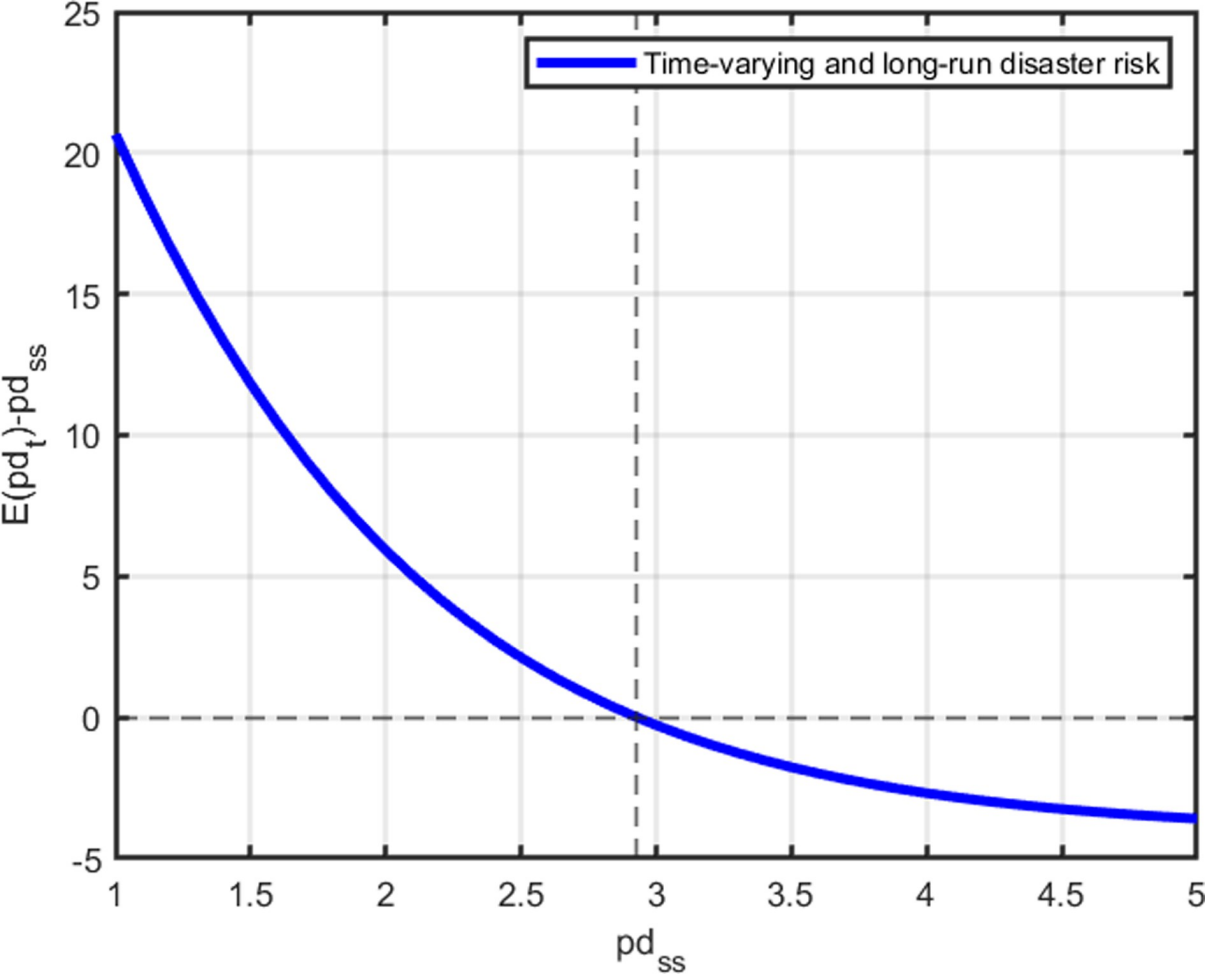
The steady state of the log price-dividend ratio.

The proof of proposition 5 is shown in Appendix E in S1 Appendix. Fig 6 illustrates how to solve a fixed point problem to find the steady state of log price-dividend ratios under the underlying parameters. We have found *pd*_*ss*_ when its assumed value equals its unconditional mean (*E*(*pd*_*t*_)).

**Fig 7:**
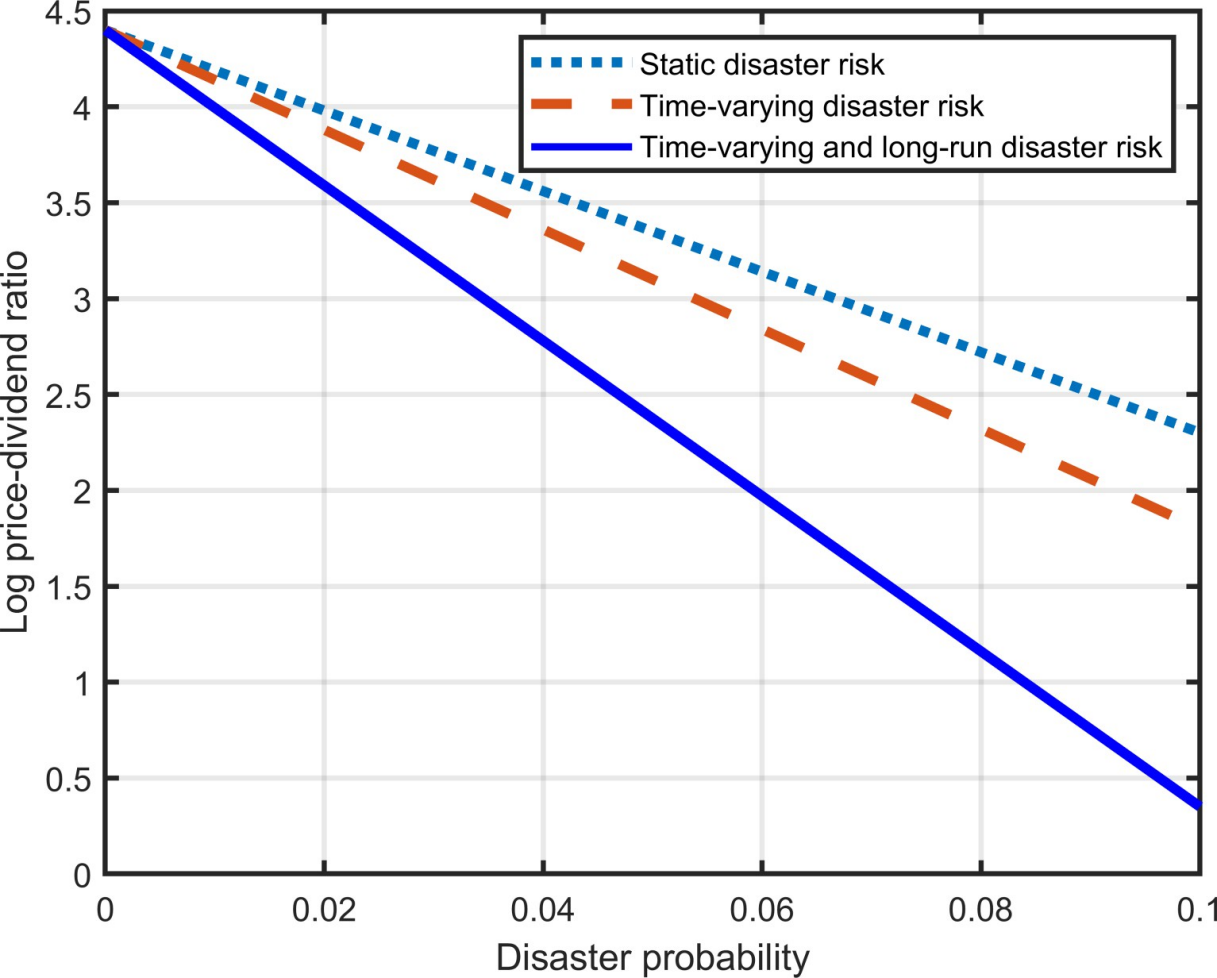
Influence of disaster probability on log price-dividend ratio in different disaster models.

Fig 7 presents the effect of disaster probability on the log price-dividend ratio in different disaster models. Although disaster risk is modeled differently, the log price-dividend ratio is always a monotonically decreasing function of disaster probability. The dashed line is below the dotted line. It is steeper than the dotted line, indicating that substituting time-varying disaster risk for static disaster risk in theoretical modeling enables the model to generate a lower log price-dividend ratio that is more sensitive to disaster probability. The solid line is below the dashed line. It is steeper than the dashed line, indicating that incorporating long-run disaster risk into the traditional time-varying disaster risk model can further reduce the log price-dividend ratio and enhance the sensitivity of the log price-dividend ratio to disaster probability.

**Fig 8:**
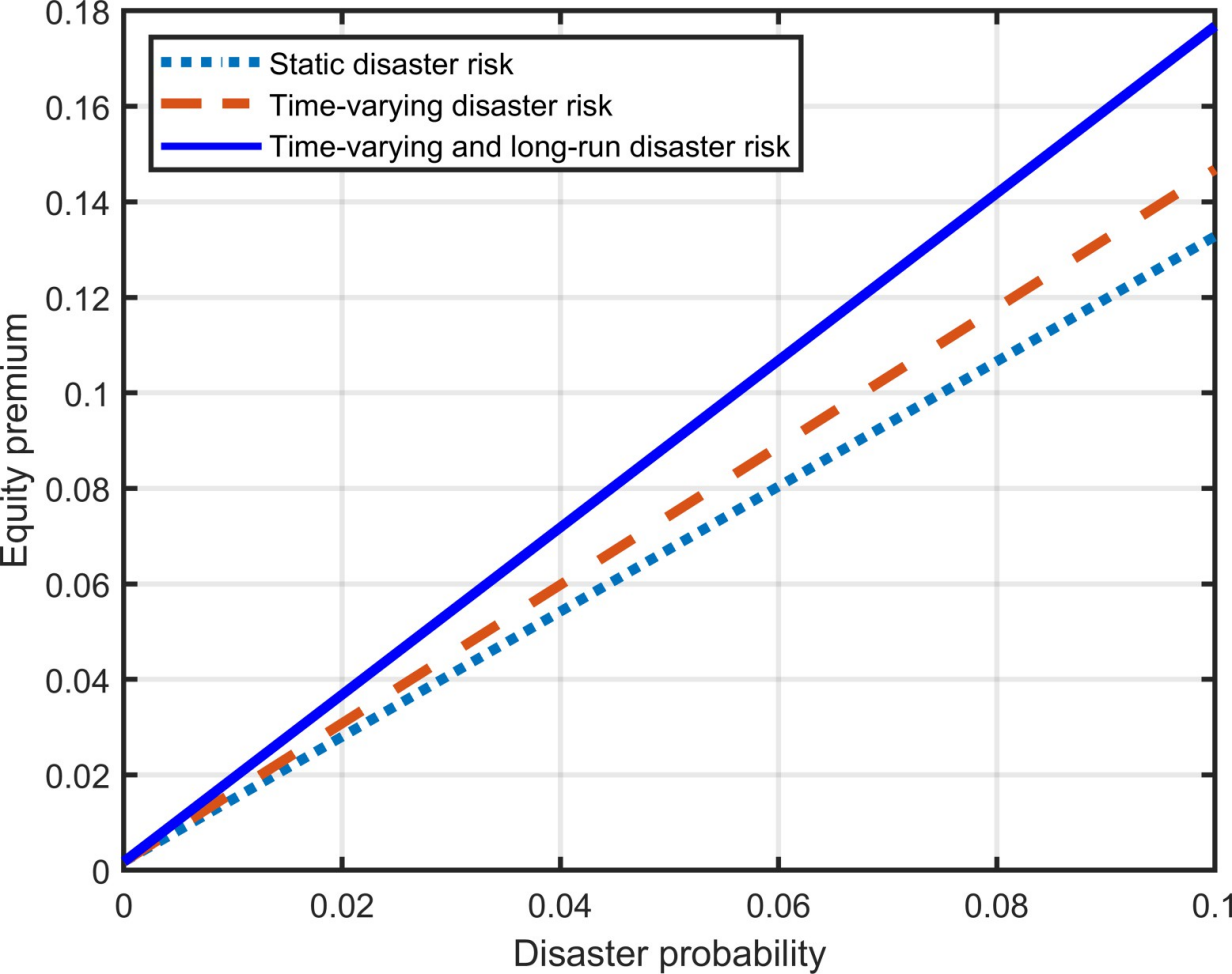
Disaster probability and the equity premium.

The corresponding relationship between equity premium and disaster probability in different disaster models is shown in Fig 8. When the disaster probability is zero, all three types of models are simplified to the standard diffusion model without disaster risk. Thus, three straight lines have an intersection. The risk premium represented by this intersection is derived from standard diffusion risk. It is rational in economics that the return people require for holding equity increases with the probability of disasters. However, in different disaster models, there are significant differences in the equity premium caused by the same disaster probability. Among them, the equity premium of the static disaster risk model (dotted line) is the smallest, the equity premium of the time-varying disaster risk model (dashed line) is second, and the equity premium of the time-varying disaster risk model, including long-run disaster risk (solid line), is the largest. The slope of the dashed line is greater than that of the dotted line, and the slope of the solid line is greater than that of the dashed line. The difference between the solid and dashed lines represents the equity premium due to long-run disaster risk and shows that it is the main component of the total risk premium.

**Fig 9:**
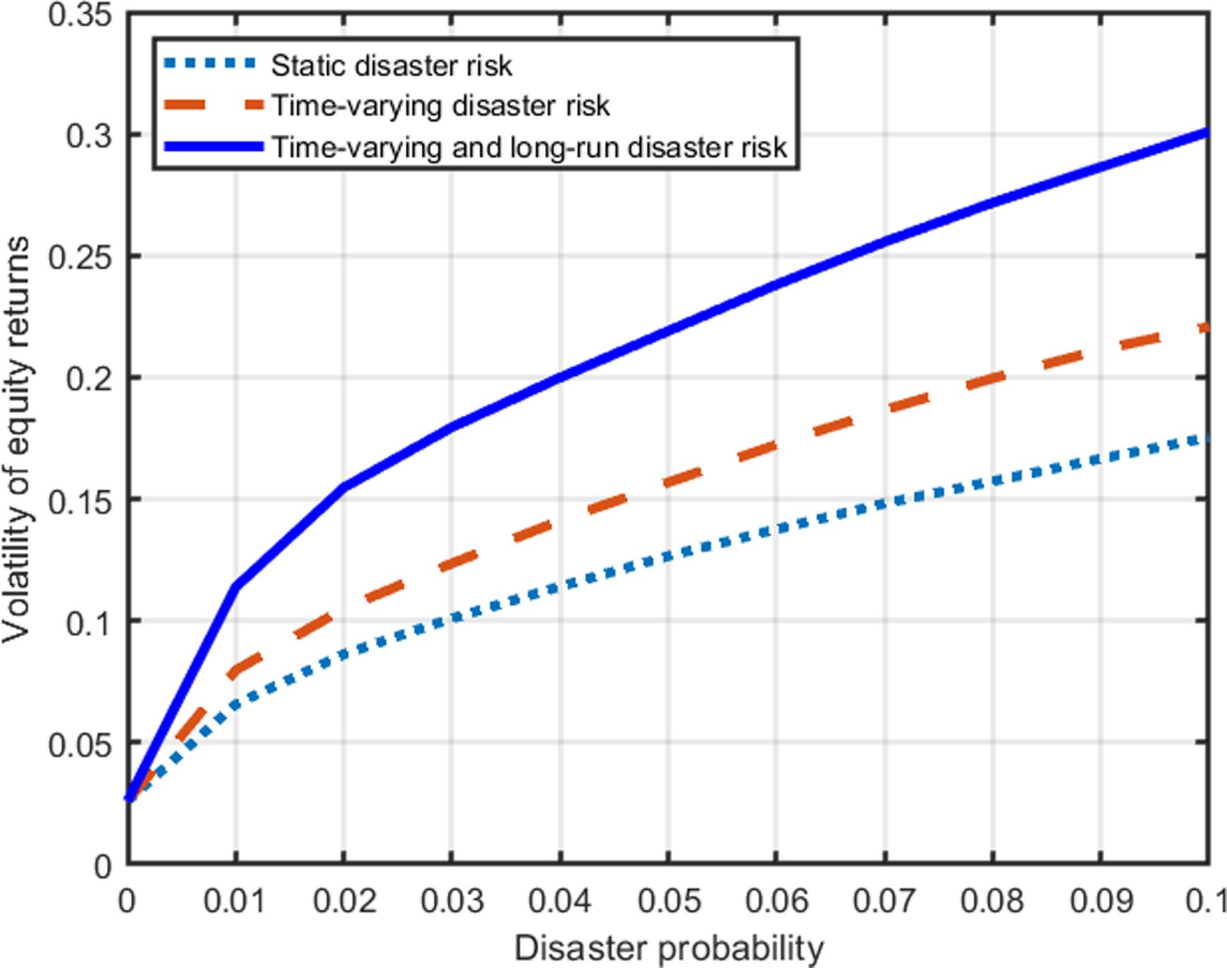
Disaster probability and volatility of equity returns.

How does equity volatility relate to disaster probability in our model? Fig 9 shows that equity volatility is a concave and increasing function of the disaster probability. When the probability of disasters is close to zero, the volatility of equity returns for all three models is close to that of the dividend in non-disaster times (Φ*σ*). Long-run disaster risk not only significantly increases equity premiums but also significantly increases the volatility of equity returns. That is, an increase in the equity premium is accompanied by an increase in the volatility of equity returns. It is worth noting that equity premium increases linearly with the disaster probability, while the volatility of equity returns increases with a square root.

**Fig 10:**
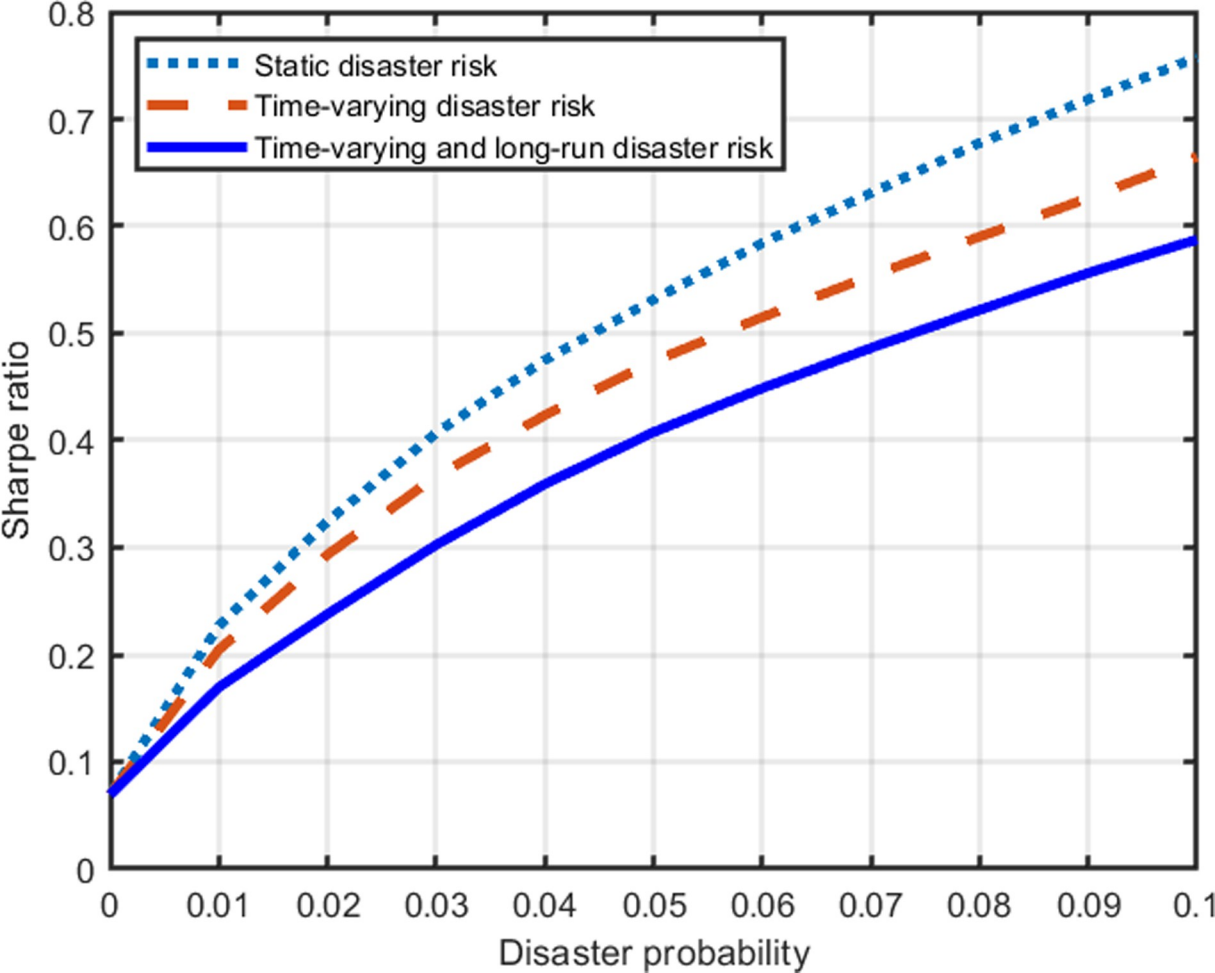
Disaster probability and the Sharpe ratio.

The Sharpe ratio equals the equity premium divided by the volatility of equity returns and measures the premium per unit of risk. The impact of disaster probability on the Sharpe ratio is shown in Fig 10.

## Calibration and simulation

Since we have analyzed in detail how the three types of disaster models differ in understanding asset returns, we next focus on matching these models to real data. Our model measures time in years and the parameter values are supplied by this.

### Calibration

Table 1 has presented the descriptive statistics of rare economic disasters. In our model, the constant term in the disaster probability process, *θ*, determines the probability of disasters when *ϕ* is fixed; the larger *θ*, the higher the average probability of disasters. Because of *E*(*p*_*t*_) = *θ*/(1-*ϕ*), the parameter *θ* can be calculated. Also, since the disaster size is assumed to obey a gamma distribution, as shown in Eq (9), we can deduce *λ* and *l* under the premise of knowing the mean and standard deviation of disaster sizes. Part A of Table 2 gives the values of disaster parameters (*λ*, *l*, and *θ*). In Part B of Table 2, we calibrate the standard parameters (*δ*, *γ*, *ϕ*, and Φ) with reference to Wachter (2013). From Eq (4), it can be seen that the volatility of consumption growth during the non-disaster period in this paper is not only affected by the short-run shock (*σε*_*t*+1_) but also by time-varying disaster probability (*p*_*t*_). Therefore, we set *σ* to a lower value than the value of this parameter in the traditional time-varying disaster risk model (0.02).27.36x10-4

**Table 2:** Parameters for simulation.

Part A: Disaster parameters		
*λ*	Distribution parameter of disaster sizes	4.05
*l*	Distribution parameter of disaster sizes	22.21
*θ*	Constant term of disaster probability process	27.36 × 10^−4^
Part B: Standard parameters		
*δ*	Subjective discount factor	0.988
*γ*	Relative risk aversion	3.0
*ϕ*	Speed of mean reversion of disaster probability process	0.92
Φ	Leverage	2.6
*σ*	Volatility of consumption growth without disaster risk	0.01

### Simulation results

Table 3 shows moments from the simulation data of these models and moments from the long historical time series (1870–2006) of the United States. Column 2 in Table 3 shows the target values of asset-pricing statistics. These targets are the mean and standard deviation of consumption growth, average equity premium, the mean and standard deviation of equity returns, the standard deviation of dividend growth, the Sharpe Ratio, and the mean and standard deviation of the risk-free rate. Column 3 in Table 3 refers to the static disaster risk model, in which the disaster probability is constant (i.e., *σ*_*p*_ = 0). The model does not include long-run disaster risk, so we set *τ* equal to zero. Given the calibrated parameters in Table 2, the model turns out to require a mean of consumption growth in non-disaster times, *μ*, of 0.025 to fit the mean of consumption growth (1.85%). The results show that the static disaster risk model seriously underestimates the equity premium, the volatility of equity returns, and the volatility of the risk-free rate. It is implausible that the volatility of the risk-free rate is equal to zero. The standard deviation of equity returns is equal to the standard deviation of dividend growth, which is also inconsistent with empirical evidence. Thus, the static disaster risk model is insufficient to characterize asset returns. Extensive literature shows that time-varying disaster risk models outperform static disaster models in understanding asset returns. Column 4 in Table 3 shows results with the model that includes only time-varying disaster risk. So, *σ*_*p*_ is set to be greater than zero, and *τ* is set to zero. Specifically, *σ*_*p*_ is set to 0.067 with reference to Wachter [8]. The value of *μ* in column 4 equals that in column 3. Overall, replacing the static disaster risk (Column 3) with the time-varying disaster risk (Column 4) generates moderate improvements in the results.

**Table 3:** Moments (annual).

	U.S. Data	Model		
		*S only*	*TV only*	*TV*&*LR*
*σ*_*p*_	-	0	0.067	0.067
*Τ*	-	0	0	-0.13
*Μ*	-	0.025	0.025	0.030
*pd_ss_*	-	3.65	3.56	2.97
Mean Consumption Growth (%)	1.85	1.85	1.85	1.85
Std Dev Consumption Growth (%)	3.60	3.81	3.94	3.97
Mean Equity Premium (%)	6.28	4.56	5.40	6.43
Mean Equity Return (%)	8.27	7.08	7.89	8.91
Std Dev Equity Return (%)	18.66	9.92	13.17	19.48
Std Dev Dividend Growth (%)	10.60	9.92	10.32	10.43
Sharpe Ratio	0.34	0.46	0.41	0.33
Mean Risk-free Rate (%)	1.99	2.52	2.47	2.48
Std Dev Risk-free Rate (%)	4.82	0.00	1.00	1.40

Notes: Data from Barro and Ursúa [2]. Here, *S only* stands for the static disaster risk model; *TV only* is the time-varying disaster risk model; *TV*&*LR* stands for the model with time-varying and long-run disaster risk.

Moments for a model with time-varying and long-run disaster risk are given in column 5 of Table 3. The parameter *σ*_*p*_ in column 5 is set to 0.067 to facilitate a comparison of the results in columns 5 and 4. It can be seen from Eq (4) that *τ* ≠ 0 means that consumption growth contains a long-run ingredient and that *τ* < 0 means that the negative impact of the long-run ingredient on consumption growth increases with the increase of disaster probability. Then, a key question is what value should *τ* take? From historical documents, it is difficult for us to find an answer to this question. Next, we try to determine the value of *τ* by matching it with the classic long-run risk model. In the classic long-run risk models, the long-run component (*z*_*t*_) is modeled as highly persistent AR(1) processes:

$$z_{t+1}=\rho z_{t}+\sigma_{z}\varepsilon_{z,t+1},$$ (19)

where *ε*_*z*,*t*+1_ ∼ *N*(0,1), and *E*(*z*_*t*_) = 0. Colacito and Croce [38] take the values of *ρ* and *σ*_*z*_ in Eq (19) to be 0.985 and 2.62 × 10^−3^, respectively. The unconditional variance of *z*_*t*_ is equal to 2.30 × 10^−4^. Based on Eq (7), an expression for the unconditional variance of the long-run component in this paper can be written: $\frac{\theta {\left(\tau \sigma_{p}\right)}^{2}}{\left(1-\phi \right)\left(1-\phi^{2}\right)}$. Assuming $\frac{\theta {\left(\tau \sigma_{p}\right)}^{2}}{\left(1-\phi \right)\left(1-\phi^{2}\right)}=2.30\times 10^{-4}$ and knowing *θ*, *σ*_*p*_ and *ϕ*, *τ* = −0.48 can be deduced. We set the value of *τ* in column 5 to -0.13 to prevent the model from generating an excessively high equity premium. Unlike the long-run ingredient in classic literature [29, 38, 40], which has no clear economic connotation, the long-run ingredient in this paper is a linear function of disaster probability (see Eq (7)). The *μ* in column 5 is larger than in column 4 to offset the effect of *τp*_*t*_ on consumption growth. The results in column 5 of Table 3 suggest that long-run disaster risk plays an important role in explaining asset returns. Comparing column 5 and column 4 in Table 3, it can be seen that the inclusion of the long-run disaster risk in traditional time-varying disaster risk models can bring a higher equity premium, a higher equity return, a higher equity return volatility, a lower Sharpe ratio, and a higher risk-free rate volatility. The above comparison shows that not only are long-run risk and disaster risk complementary in explaining asset returns but they may be driven by common factors such as disaster probability. Although Barro and Jin [28] also point out that rare disaster models and long-run risk models are complementary, the disaster probability is static in their model, and the long-run risk still lacks a connotation. In Barro and Jin [28], disaster risk and long-run risk affect asset returns independently in their ways.

It can be seen from the above that the consumption-based capital asset pricing model established in this paper can well explain asset returns. So how do rare economic disasters affect asset returns in this article? In theory, a consumption-based capital asset pricing model might perfectly explain asset returns only if all risks of consumption growth are priced in. According to the existing literature, risks to consumption growth include short-run risks and long-run risks. In this paper, rare economic disasters affect consumption growth through two channels: first, random rare economic disasters are one of the sources of short-run risks for consumption growth; secondly, the probability of rare economic disasters with high first-order autocorrelation is a source of long-run risks for consumption growth. Eq (4) shows that when no rare economic disaster occurs in period *t* + 1, the expected growth rate of consumption in this paper is *μ* + *τp*_*t*_, which is a constant in the traditional model. In practice, even if there is no rare economic disaster in the future, people’s expected consumption growth rate will change with the probability of a rare economic disaster in the future. Furthermore, the agent has recursive preferences in our model, and the agent’s need to address risk ahead of time leads to the pricing of expected short-run and long-run risks.

### Implied disaster probability

We combine the S&P 500 price-earnings ratio with the models in this subsection to derive the implied disaster probability. Eq (18) shows that the price-dividend ratio is a monotonically decreasing function of disaster probability (*A* < 0). Therefore, the implied disaster probability can be calculated if the price-dividend ratio is known. Wachter [8] points out that for long-time series data, the price-earnings ratio from the data should be used to match the price-dividend ratio of the theoretical model. Robert J. Barro’s website (https://scholar.harvard.edu/barro/data_sets) provides the S&P 500 price-earnings ratio from 1880 to 2021 and the method for calculating the price-earnings ratio. When calculating the implied disaster probability, we set the mean-removed price-earnings ratio in the data equal to the mean-removed price-dividend ratio in the model. When the disaster probability is negative, it is set to zero.

**Fig 11:**
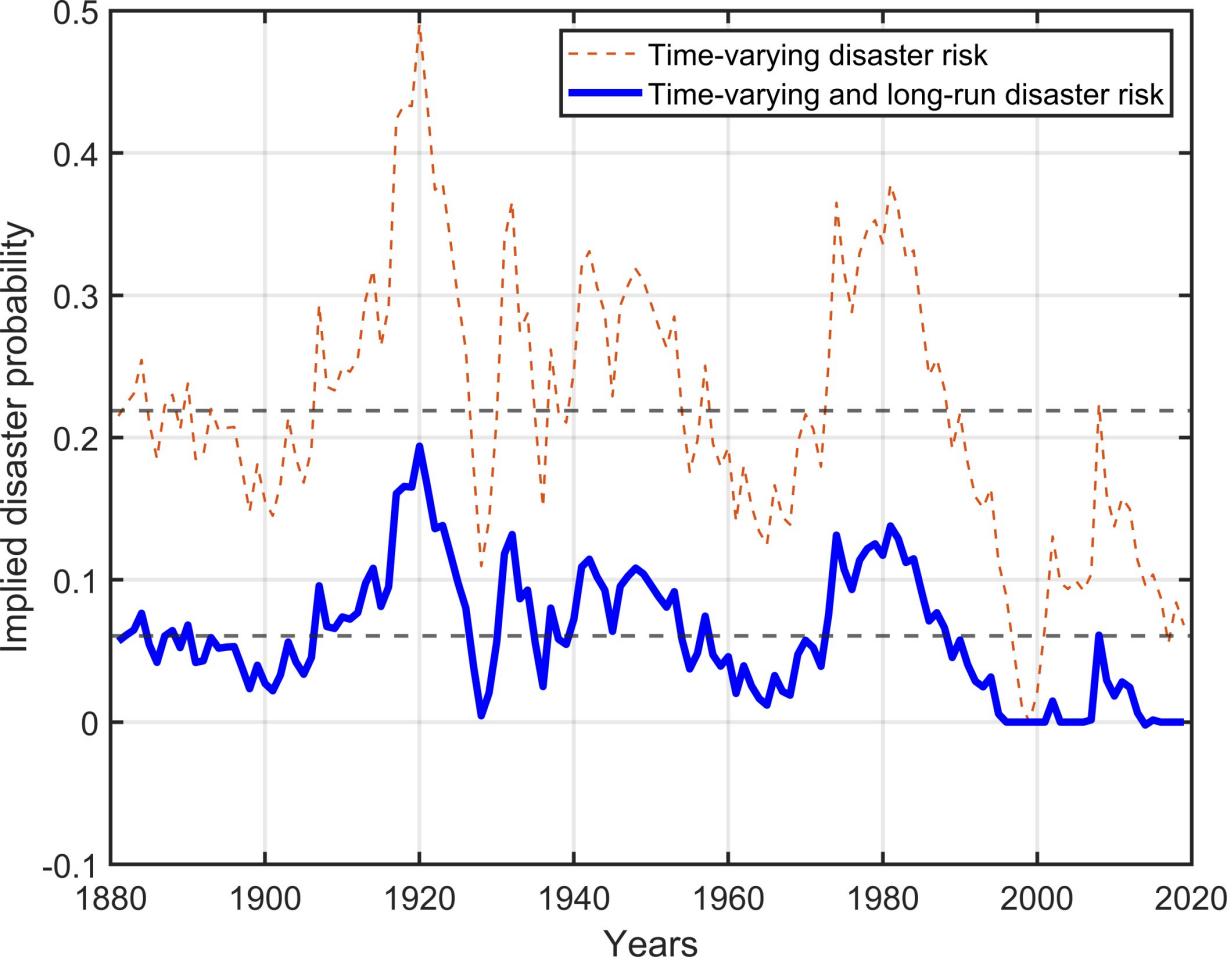
Implied disaster probability in the United States. The horizontal dashed lines represent the means of the two curves, respectively.

It can be seen from Table 1 that the average probability of rare economic disasters is 0.0342. According to the calibrated parameters (*θ*, *ϕ*, and *σ*_*p*_), we implicitly assume that the standard deviation of disaster probability is 0.0316. Fig 11 depicts the implied probability of disasters for the United States calculated using P/E data. For the time-varying disaster model (dashed line), the mean and standard deviation of implied disaster probability are 0.2189 and 0.0943, respectively; for our model with time-varying and long-run disaster risk (solid line), they are 0.0607 and 0.0436, respectively. The solid line in Fig 11 better reflects the historical real disaster probability. These results also indirectly support our assumption that the long-run ingredient in consumption growth rates is a function of disaster probability.

### Predictability

Bansal and Yaron [29] argue that there is a small but persistent component of U.S. consumption growth over the past century, which they call long-run risk. The long-run risk in consumption growth rates does not conflict with empirical evidence that consumption growth is close to a random walk. Long-run risk, while only explaining a small part of consumption volatility, explains a large part of asset returns and their volatility under recursive preference. Estimating long-run risk in consumption growth rates is a big challenge. Colacito and Croce [40] refer to Bansal et al. [36], using risk-free rates, consumption-output ratios, lagged consumption growth, lagged price-dividend ratios, and default premium to systematically estimate the long-run ingredient of consumption growth; the results show that the long-run ingredient does exist.

The ability of disaster probability to predict macroeconomic growth is a common feature of various disaster models. Although this feature contradicts the unpredictability of consumption growth pointed out by empirical evidence, the importance of disaster models for understanding asset returns is unquestionable. Therefore, we need a rare disaster model that can mitigate the above conflicts efficiently. Most existing disaster models lack modeling of the long-run ingredient of economic growth. In our model, we assume that the long-run ingredient in consumption growth rates is a linear function of the disaster probability. This is an innovation. Next, we will prove that our assumption about the consumption process is closer to the dynamics of actual consumption data than those in traditional time-varying disaster risk models. That is, the predictability of consumption growth by disaster probability is significantly reduced in our model.

In reality, the probability of disaster is unobservable. Eq (18) shows that the log price-dividend varies linearly with the probability of a disaster. Therefore, we can use the log price-dividend ratio instead of disaster probability to predict consumption growth. Long-horizon consumption growth is regressed on the lagged log price-dividend ratio in simulated data and actual data for the United States from 1880 to 2021. The regression equation is as follows:

$$\sum_{j=1}^{h}\;\Delta c_{t+j}=\beta_{0}+\beta_{1}pd_{t}+\epsilon_{t},\;h\geq 1.$$ (20)

As shown in Panel A of Table 4, in U.S. data, while long-horizon consumption growth is unpredictable, short-horizon consumption growth is predictable to a certain extent. At the one-year horizon, *β*_1_ is 0.01, *R*^2^ is 0.03, and *T*-statistic is equal to 2.11; otherwise, the regression coefficients are insignificant, and the maximum *R*^2^ does not exceed 0.01. In Panel B of Table 4, consumption growth is regressed on the lagged log price-dividend ratio in data simulated from the time-varying disaster risk model. In Panel C of Table 4, the results refer to our model. Unsurprisingly, in both models, the price-dividend ratio significantly predicts consumption growth. In each horizon, the *R*^2^ in Panel C is close to that in Panel B, and the *T*-statistic in Panel C is close to that in Panel B. However, the *β*_1_ in Panel C is significantly smaller than in Panel B on each horizon. Obviously, in terms of unpredictability in consumption growth rates, the data simulated from our model is more consistent with the actual data. This is because the long-run disaster risk has a much smaller impact on consumption growth than on the log price-dividend ratio, reducing consumption growth’s predictability.

**Table 4:** Long-horizon regressions: Consumption growth.

	Horizon in Years					
	1	2	4	6	8	10
Panel A: U.S. Data						
*β*_1_	0.01	0.01	-0.00	-0.01	0.01	0.01
*R* ^2^	0.03	0.01	0.00	0.00	0.00	0.00
*T* − *stat*	2.11	0.88	-0.29	-0.38	0.31	0.63
Panel B: Time-varying disaster risk						
*β*_1_	0.07	0.13	0.22	0.32	0.36	0.41
*R* ^2^	0.13	0.22	0.28	0.32	0.30	0.29
*T* − *stat*	4.73	6.42	7.53	8.25	7.67	7.44
Panel C: Time-varying and long-run disaster risk						
*β*_1_	0.04	0.07	0.12	0.16	0.19	0.21
*R* ^2^	0.12	0.22	0.32	0.33	0.30	0.27
*T* − *stat*	4.51	6.42	8.21	8.33	7.75	7.11

Notes: U.S. consumption growth data is from Robert J. Barro’s website (https://scholar.harvard.edu/barro/data_sets), and the log price-dividend ratio data for the U.S. is from Robert J. Shiller’s website (http://www.econ.yale.edu/∼shiller/data.htm).

How does long-run disaster risk in this paper affect the predictability of equity premiums? The long-horizon equity premium is regressed on the lagged log price-dividend ratio in simulated data and actual data for the United States from 1991 to 2021. The regression equation is as follows:

$$\sum_{j=1}^{h}\;r_{e,t+1}-r_{f,t+1}=\beta_{0}+\beta_{1}pd_{i,t}+\epsilon_{t},\;h\geq 1.$$ (21)

Panel A of Table 5 shows that the regression coefficient on the log price-dividend ratio (*β*_1_) is significantly less than zero: a high disaster probability corresponds to a low log price-dividend ratio (see Eq (18)) and thus also predicts a high future expected equity premium. *β*_1_ is -0.13 at the one-year horizon, decreasing to -0.86 at the ten-year horizon. Meanwhile, *R*^2^ is 0.09 at the one-year horizon, rising to 0.43 at the ten-year horizon. Panel B reports the moments of the model with time-varying disaster risk. In panel B, the regression coefficients are insignificant at both the one- and two-year horizons; this suggests that the log price-dividend ratio cannot predict the short-horizon equity premium. Panel C reports the results of our model with time-varying and long-run disaster risk. In panel C, the regression coefficient is significant on each horizon; *β*_1_ is -0.12 at the one-year horizon, decreasing to -0.75 at the ten-year horizon; *R*^2^ is 0.05 at the one-year horizon, rising to 0.22 at the ten-year horizon. The results in plane C are closer to those in plane A than those in plane B because significantly reduce the price-dividend ratio. Moreover, *β*_1_ in plane C divided by *β*_1_ in plane B equals 2.4 at the one-year horizon, decreasing to 1.15 at the ten-year horizon; this is because the long-run disaster risk not only increases the total amount of risk in the economy but also changes the distribution of equity premiums over time. With recursive preference, agents are risk-averse to future risk; with the extension of the horizon, the impact of the long-run disaster risk on equity premiums gradually weakens.

**Table 5:** Long-horizon regressions: Equity premium.

	Horizon in Years					
	1	2	4	6	8	10
Panel A: U.S. Data						
*β*_1_	-0.13	-0.23	-0.33	-0.48	-0.64	-0.86
*R* ^2^	0.09	0.17	0.23	0.30	0.38	0.43
*T* − *stat*	-2.62	-2.87	-3.64	-4.80	-5.82	-5.67
Panel B: Time-varying disaster risk						
*β*_1_	-0.05	-0.14	-0.29	-0.50	-0.60	-0.65
*R* ^2^	0.00	0.02	0.04	0.10	0.12	0.11
*T* − *stat*	-0.81	-1.62	-2.58	-4.05	-4.32	-4.01
Panel C: Time-varying and long-run disaster risk						
*β*_1_	-0.12	-0.24	-0.33	-0.48	-0.63	-0.75
*R* ^2^	0.05	0.10	0.11	0.15	0.20	0.22
*T* − *stat*	-2.65	-3.85	-4.10	-5.04	-5.85	-6.13

Notes: Constrained by data availability, we calculated the price-dividend ratio and equity premium in the United States from 1991 to 2021. Data on S&P 500 and dividend yields from Global Financial Data (GFD) by Siblis Research. Yields on 3-month T-bills and consumer price index (CPI) were collected from Federal Reserve Economic Data (FRED).

## Conclusions

To improve traditional rare disaster models, we redefine macroeconomic disasters and propose a novel rare disaster model with time-varying and long-run disaster risk. Using the technique of linearity-generating processes, the model is tractable, and all asset prices are solved in closed form. Our main conclusions are as follows.

Firstly, incorporating long-run disaster risk into traditional rare disaster models can increase the volatility of asset returns and the sensitivity of asset returns to disaster risk. Secondly, our model with time-varying and long-run disaster risk matches the U.S. data better than traditional rare disaster models with only static or time-varying disaster risk. In our model, disaster risk affects asset returns not only through a short-run channel (the time-varying disaster risk) but also through a long-run channel (the long-run disaster risk).

Our model not only facilitates the analysis of the mechanism by which disaster risk affects asset returns but also provides a new potential channel for disaster risk to affect asset returns. It also enriches the connotation of long-run risk and strengthens the inherent connection between long-run risk models [29] and traditional rare disaster models [8]. Since the disaster probability is unobservable, it is unrealistic to directly prove our model’s fundamental assumption that the long-run ingredient of consumption growth is a function of time-varying disaster probability. Providing more evidence for this assumption may be a future research direction. Extending our theoretical framework to pricing other asset classes (e.g., bonds, exchange rates, and options) is also a future research direction.

## Supporting information

S1 Data (XLSX)

S1 Appendix (DOCX)
