# Supplementary material for: Asset pricing with long-run disaster risk
Source: PLoS One. 2023 Jun 27;18(6):e0287687. doi: 10.1371/journal.pone.0287687 (PMC10298804; doi:10.1371/journal.pone.0287687)
Supplement: S1 Appendix — (DOCX) [file pone.0287687.s002.docx]

Appendices

**Appendix A: Proof of Proposition 1.**

The logarithm of $U_{t}$ is $\log U_{t}=\frac{\log\left\{ \left( 1-\delta\right)\left( C_{t}^{i} \right)^{1-1/\psi}+\delta E_{t}\left[ \left( U_{t+1}^{i} \right)^{1-\gamma} \right]^{\frac{1-1/\psi}{1-\gamma}} \right\}}{1-1/\psi}$. Using L'Hospital's rule, the limit of it when $\psi=1$ is $\lim_{\psi\to1} \frac{\log\left\{ \left( 1-\delta\right)\left( C_{t}^{i} \right)^{1-1/\psi}+\delta E_{t}\left[ \left( U_{t+1}^{i} \right)^{1-\gamma} \right]^{\frac{1-1/\psi}{1-\gamma}} \right\}}{1-1/\psi}=\left( 1-\delta\right)\log C_{t}+\frac{\delta}{1-\gamma}\log E_{t}\left[ e^{\left( 1-\gamma\right)\log U_{t+1}} \right].$

**Appendix B: Proof of Proposition 2.**

According to assumption 1, $E_{t}\left( e^{\left( 1-\gamma\right)\zeta_{t+1}} \right)=E_{t}\left[ e^{\left( 1-\gamma\right)\varpi_{t+1}} \right]E_{t}\left[ e^{\left( \gamma-1 \right){\vartheta_{t+1}N}_{t+1}} \right]$ and $E_{t}\left( e^{\left( 1-\gamma\right)\varpi_{t+1}} \right)=e^{\left( 1-\gamma\right)v_{t}+\frac{\left( 1-\gamma\right)^{2}}{2}\chi_{t}^{2}}. N_{t+1}$ follows a Bernoulli distribution as shown in Eq.(5), so $E_{t}\left[ e^{\left( \gamma-1 \right)\vartheta_{t+1}N_{i,t+1}} \right]=p_{t}e^{\left( \gamma-1 \right)\vartheta_{t+1}}+1-p_{t}\approx e^{\left( E_{t}\left[ e^{\left( \gamma-1 \right)\vartheta_{t+1}} \right]-1 \right)p_{t}}.$ Combining Eq.(9), $E_{t}\left[ e^{\left( \gamma-1 \right)\vartheta_{t+1}N_{i,t+1}} \right]=e^{\left[ \left( 1-\frac{\gamma-1}{\iota} \right)^{-\lambda}-1 \right]p_{t}}$.

**Appendix C: Proof of Proposition 3.**

Eq.(3) can be rewritten as $\log U_{t}-\log C_{t}=\frac{\delta}{1-\gamma}\log E_{t}\left[ e^{\left( 1-\gamma\right)\left( \log U_{t+1}-\log C_{t} \right)} \right],$ where $\log U_{t}-\log C_{t}=y_{t}$ and $\log U_{t+1}-\log C_{t}=\zeta_{t+1}$. Based on Eq.(11), it is easy to get Eq.(12).

**Appendix D: Proof of Proposition 4.**

If we assume that $y_{t}=L_{0}+{Lp}_{t}$, then $\zeta_{t+1}=L_{0}+\mu+L\theta+\left( L\phi+\tau\right)p_{t}+\sigma\varepsilon_{t+1}+L\sigma_{p}\sqrt{p_{t}}\varepsilon_{p,t+1}-\vartheta_{t+1}N_{t+1}$. Using assumption 1, $v_{t}=L_{0}+\mu+L\theta+\left( L\phi+\tau\right)p_{t}$ and $\chi_{t}^{2}=\sigma^{2}+\left( L\sigma_{p} \right)^{2}p_{t}$. Eq.(11) implies that$y_{t}=\delta\left( L_{0}+\mu+L\theta\right)+\frac{\delta\left( 1-\gamma\right)}{2}\sigma^{2}+\delta\left[ \frac{1-\gamma}{2}\left( L\sigma_{p} \right)^{2}+\left( L\phi+\tau\right)+\frac{\left( 1-\frac{\gamma-1}{\iota} \right)^{-\lambda}-1}{1-\gamma} \right]p_{t}.$ Therefore, the following two equations can be given: $\delta\left( L_{0}+\mu+L\theta\right)+\frac{\delta\left( 1-\gamma\right)}{2}\sigma^{2}=L_{0}$ and $\delta\left[ \frac{1-\gamma}{2}\left( L\sigma_{p} \right)^{2}+\left( L\phi+\tau\right)+\frac{\left( 1-\frac{\gamma-1}{\iota} \right)^{-\lambda}-1}{1-\gamma} \right]=L$. It is easy to calculate $L_{0}$ and $L$.

**Appendix E: Proof of Proposition 5.**

Solving the Euler equation $\left( E_{t}\left[ e^{m_{t+1}+r_{e,t+1}} \right]=1 \right)$ based on the assumption that $pd_{t}=$ $A_{0}+Ap_{t}$ can yield the following equations: $\log\left[ \delta\left( e^{pd_{ss}}+1 \right) \right]-\mathcal{l}pd_{ss}+\left( \mathcal{l}-1 \right)A_{0}+\left( \Phi-1 \right)\mu+\mathcal{l}\theta A-\frac{\left( 1-\gamma\right)^{2}\sigma^{2}}{2}+\frac{\left( \Phi-\gamma\right)^{2}\sigma^{2}}{2}=0,\frac{\mathcal{l}^{2}\sigma_{p}^{2}}{2}A^{2}+\left[ \mathcal{l}\left( 1-\gamma\right)L\sigma_{p}^{2}+\mathcal{l}\phi-1 \right]A+\left( \Phi-1 \right)\tau+{\left( 1-\frac{\gamma-\Phi}{\iota} \right)^{-\lambda}-\left( 1-\frac{\gamma-1}{\iota} \right)}^{-\lambda}=0.$ It is easy to calculate $A_{0}$ and $A$.
